# Bedside pleuroscopy in Taiwan : a great vision for critically-ill patients and intensivists

**DOI:** 10.7603/s40681-016-0013-5

**Published:** 2016-08-12

**Authors:** Hean Ooi

**Affiliations:** 1 Department of Preventive Medicine, Buddhist Tzu Chi General Hospital, Taichung Branch, 427 Taichung, Taiwan; 2 Division of Chest and Critical Care Medicine, Buddhist Tzu Chi General Hospital, Dalin Branch, 622 Chiayi, Taiwan; 3 School of Medicine, Tzu Chi University, 970 Hualien, Taiwan; 4 Department of Medical Imaging and Radiological Sciences, Central Taiwan University of Science and Technology, No. 11, Buzih Lane, 406 Taichung, Taiwan; 5 Department of Medical Research, China Medical University Hospital, China Medical University, 406 Taichung, Taiwan

**Keywords:** Bedside, Intensive Care Unit (ICU), Pleuroscopy, Taiwan

## Abstract

Bedside pleuroscopy can be used in daily practice by medical pulmonologists if a patient cannot tolerate either general anesthesia or being moved to an operating or endoscopy room due to their critical condition. It is a simple and safe technique that rarely has complications. The aim of this review is to summarize recent literatures about bedside pleuroscopy and share our experiences with using it in Taiwan.

## 1. Introduction

Pleuroscopy is an indispensable tool for the diagnosis and treatment of pleural disease. The instrument is easy to manipulate because the endoscope is of a similar design as a standard flexible bronchoscope. Previous studies have shown the efficacy of pleuroscopy, particularly in cases with pleural effusion [1-3]. Yet it is a procedure seldom used on critically-ill patients and in intensive care unit (ICU) bedsides, so here in we have reviewed bedside pleuroscopy and share our experiences with using it in Taiwan.

## 2. The development of pleuroscopy in Taiwan- Flexible pleuroscopy

The earliest use of a fexible bronchoscope as a fiberoptic pleuroscope was done in 1975 in America [4, 5]. At the time pulmonologists dubbed the technique “pleuroscopy”. Since then, flexible bronchoscopes have been introduced to hospitals around the world, especially where no suitable tools were available for the diagnosis or treatment of pleural disease [6, 7]. Recently, pleuroscopies performed under local anesthesia using a chest tube with a flexible fiberoptic bronchoscope have been reported [8, 9]. This technique has been used in many countries included developed and developing ones (Japan, China, Egypt, and Taiwan, for example) [8-10]. This so-called flexible fiberoptic pleuroscopy may be able to provide a diagnosis of exudative pleural effusions when other less invasive procedures fail to do so [11, 12].

This flexible pleuroscopy under local analgesia using a flexible bronchoscope is a simple procedure performed at the bedside and suitable for those critically-ill patients who cannot be moved to an operating or endoscopic room [13, 14]. At our institution, respiratory physicians have been performing pleuroscopies with a flexible bronchoscopies for over 5 years (since 2010) in the ICU [8,9]; however, there are still some limitations: They are as follows:

1. A pleuroscopy with a flexible bronchoscopy is more difficult to manipulate within the pleural cavity than within the bronchi and does not provide a good orientation within the pleural space. This procedure also has a long learning curve and needs supervision from an experienced endoscopist as well as a lot of practice [15, 16].
2. A flexible bronchoscope has a small specimen in comparison with a rigid thoracoscope. Thus, we have increased the biopsy site and during the procedure take more than 10 specimens [17, 18].
3. Damage done to the rubber shirt of the bronchoscope: we use a plastic trocar and a chest tube to protect the shirt of the bronchoscope [19, 20]. This helps to diminish the damage done to the tool. However, it must be considered that there are different facilities available in different countries or just different hospitals [21-23]. In our department, we did not have a semiflexible pleuroscope from 2010 to 2014, so we had to make do with using a flexible bronchoscope during that time for pleuroscopies at critically-ill patients’ bedsides once they were determined to require pleuroscopies [6-10].

However, the advantages of a flexible pleuroscopy are that at least it does not require extra- money to buy an additional instrument and that there is no need to worry about whether there is a suitable tool in the hospital to perform pleuroscopies [26, 27].

### 2.1. Semi-flexible pleuroscopy

A semi-flexible pleuroscope with rigid shafts and flexible tips was developed in 1978 in Japan by Takeno [28]. Today, the most commonly used semi-flexible pleuroscope was developed by Olympus Corporation in 2002 [29], with a working channel of 2.8 mm and incorporated video imaging. This semi-felxible pleuroscope was introduced into Taiwan only in 2014 after the various efforts of flexible pleursocopy performed at Taichung’s Tzu Chi Hospital [6-10]. This pleuroscope consists of a handle that is similar to a standard flexible bronchoscope and a shaft that measures 7 mm in outer diameter and 27 cm in length. The shaft is made up of two sections, a 22 cm proximal rigid portion and a 5 cm flexible distal end [30, 31]. The flexible tip is movable by a lever on the handle, which allows two-way angulation capability of 160° upward and 130° downward. It also has a 2.8-mm working channel that can accommodate biopsy forceps, needles, and other accessories and is compatible with various electrosurgical and laser procedures. The other advantage of the semi-flexible pleuroscope is that it interfaces easily with existing processors and light sources manufactured for flexible video bronchoscopy [32, 33].

## 3. Bedside pleuroscopy for critically-ill patients

**Figure 1:**
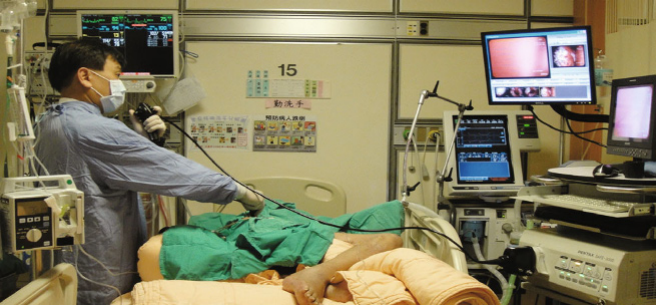

The main indication for the necessity of performing a pleuroscopy is an exudative pleural effusion with an unknown etiology [30-33]. For critically-ill patients with acute respiratory failure due to an unresolved exudative pleural effusion were challengeable [9, 10]. It is not always possible to move critically-ill patients to the operating or endoscopy room for a pleuroscopy due to their critical condition; and there is the uncertainty of the waiting time for the operation to consider too. Because the crude mortality rates are higher for intensive care unit (ICU) patients with pleural effusions than for those without pleural effusions, in 2010 we began using pleuroscopy at the bedside in the ICU to diagnose pleural effusions in patients with acute respiratory failure [Fig. 1]. This was when there were a large number of critically-ill patients that needed their pleural problems solved in the ICU in order to decrease their mortality rate [34, 35].

It is known that a standard rigid or semi-rigid thoracoscopy has several advantages over a pleuroscopy under local analgesia [35, 37], such as the ability to obtain larger biopsy specimens and better control of the bleeding. However, a thoracoscopy is a more invasive technique than a pleuroscopy that requires general anesthesia with a double-lumen endotracheal tube and selective lung ventilation [38, 39]. Therefore, a thoracoscopy has to be carried out by surgeons with a large number of operative instruments, and anesthesiologists are also needed in the surgical suite [40]. Pleuroscopy with local anesthesia is a less invasive and less expensive approach to thoracoscopy. Many studies have reported that pleuroscopy performed by pulmonologists is a safe and effective modality for the diagnosis of pleural effusions [36-40].

**Figure 2:**
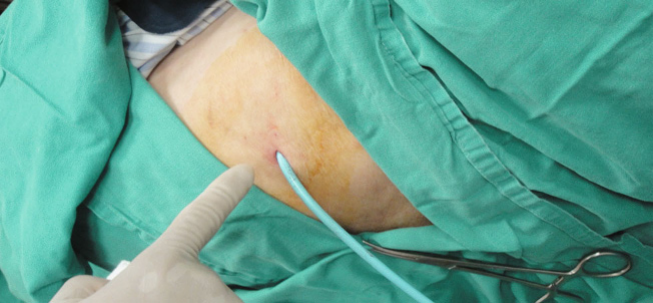

In our case in Taiwan, because of the small size of the trocar insertion wound and the small diameter of the instruments, we were able to use a pigtail 16 Fr catheter as the drainage catheter without any suture stitches. Standard chest tube insertion requires sutures and also uses tubes of a bigger size like 32 Fr [41]. To the best of our knowledge, this is the first use ever of bedside pleuroscopy in an ICU using a pigtail 16 Fr catheter [Fig. 2] for drainage without any suture stitches [6-9].

## 4. The etiology and pleural finding in ICU bedsides

The causes of most pleural effusions in critically-ill patients are secondary to malignancy or infections. The presences of malignancies (60%) and infections (36%) also have been noted in our study. We performed adhesiolysis at the time of pleuroscopy at ICU bedsides [42], as well as ensuring adequate drainage with a pigtail catheter to allow for the re-expansion of the lungs. The list of etiologies of pleural effusions is extensive; however, a bloody effusion with malignancy is still the main cause of an undiagnosed pleural effusion [43]. The endoscopic appearance of pleural lesions suggestive of a malignancy includes nodules, polypoid lesions, masses, and localized “candle wax drops” in the literature [44, 45].

Actually, most of the pleural lesions in critically-ill patients appear to be infiltrating (40%) or a combination of nodules and infiltrating (40%), but not all are nodular in appearance (20% are not) as found in previous studies [22, 23, 24]. Clinicians should be alert to this when performing a pleuroscopy for an undiagnosed pleural effusion in patients with acute respiratory failure [6-9].

## 5. Complications of bedside pleuroscopy

Major complications resulting from pleuroscopy have been reported in 0.0001 to 0.24% of patients [46], the most serious being bleeding or death. Major complications are not seen frequently,however. In our institution we had one patient who experienced CO2 narcosis during these periods, so protecting the patient’s airway and the equipped monitors was very important during the procedure. Minor complications of the procedure include subcutaneous emphysema, insignificant pneumothorax, wound pain, and postoperative fever and infection [47, 48]. All of these conditions have easily been controlled and have been self-limited [49, 50].

## 6. A great vision for critically-ill patients and intensivists

Rapid response or early management for critically-ill patients with undiagnosed exudative pleural effusion is very important. A quick clinical practice in the ICU at a patient’s bedside requires little to no waiting, and the easy to manipulated pleuroscopy has few complications. Indeed, bedside pleuroscopy is a simple and well-tolerated procedure with local analgesia. It can be used as a routine by medical pulmonologists or ICU physicians if their patients are not able to undergo general anesthesia or moving them to the operating or endoscopic room is unwise due to their critical condition. It is a great vision for all critically-ill patients and also their intensivists.
